# Discovery of a new mitochondria permeability transition pore (mPTP) inhibitor based on gallic acid

**DOI:** 10.1080/14756366.2018.1442831

**Published:** 2018-03-07

**Authors:** José Teixeira, Catarina Oliveira, Fernando Cagide, Ricardo Amorim, Jorge Garrido, Fernanda Borges, Paulo J. Oliveira

**Affiliations:** a CIQUP, Department of Chemistry and Biochemistry, Faculty of Sciences, University of Porto, Porto, Portugal;; b Center for Neuroscience and Cell Biology, University of Coimbra, UC-Biotech, Cantanhede, Portugal;; c PhD Programme in Experimental Biology and Biomedicine (PDBEB), Center for Neuroscience and Cell Biology, University of Coimbra, Coimbra, Portugal;; d III-Institute for Interdisciplinary Research, University of Coimbra, Portugal;; e Department of Chemical Engineering, School of Engineering (ISEP), Polytechnic Institute of Porto, Porto, Portugal

**Keywords:** Gallic acid, mitochondriotropic antioxidant, oxidative stress, mitochondrial dysfunction, mitochondrial permeability transition pore

## Abstract

Pharmacological interventions targeting mitochondria present several barriers for a complete efficacy. Therefore, a new mitochondriotropic antioxidant (AntiOxBEN_3_) based on the dietary antioxidant gallic acid was developed. AntiOxBEN_3_ accumulated several thousand-fold inside isolated rat liver mitochondria, without causing disruption of the oxidative phosphorylation apparatus, as seen by the unchanged respiratory control ratio, phosphorylation efficiency, and transmembrane electric potential. AntiOxBEN_3_ showed also limited toxicity on human hepatocarcinoma cells. Moreover, AntiOxBEN_3_ presented robust iron-chelation and antioxidant properties in both isolated liver mitochondria and cultured rat and human cell lines. Along with its low toxicity profile and high antioxidant activity, AntiOxBEN_3_ strongly inhibited the calcium-dependent mitochondrial permeability transition pore (mPTP) opening. From our data, AntiOxBEN_3_ can be considered as a lead compound for the development of a new class of mPTP inhibitors and be used as mPTP de-sensitiser for basic research or clinical applications or emerge as a therapeutic application in mitochondria dysfunction-related disorders.

## Graphical abstract

**Figure:**
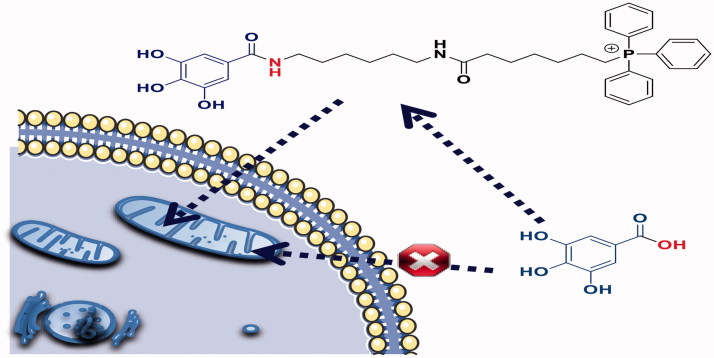

## Introduction

Increasing evidence suggests that mitochondrial dysfunction amplifies oxidative stress events playing a crucial role in different pathologies^1–4^. Therefore, mitochondria are attractive targets for several classes of molecules which are aimed to minimise organelle damage, a process involved in the pathophysiology of several diseases. Still, while the role of mitochondria in disease pathogenesis is generally recognised, achieving a targeted therapeutic effect in that organelle is not straightforward^5–7^. Mitochondrial-related diseases treatments are normally focused on maintaining tissue health using preventive measures to mitigate symptom worsening, such as the optimisation of nutrition and administration of vitamins and food supplements, along with symptom-based management^8–10^.

Epidemiological studies and associated meta-analyses suggest that long-term consumption of diets rich in plant polyphenols plays a meaningful role in the prevention and/or avoidance of oxidative-stress related events^11^. Gallic acid (3,4,5-trihydroxybenzoic acid) is a plant phenolic compound widely found in diet. Its antioxidant activity has been associated to its ability to chelate pro-oxidant transition metals (e.g. Cu and Fe), to scavenge radicals by hydrogen donation and/or electron transfer, and to inhibit lipid peroxidation processes as well as several pro-oxidant enzymes involved in reactive oxygen radical (ROS) production^12^ ^,^ ^13^.

Although gallic acid is considered to be a versatile antioxidant, its hydrophilic nature restricts its bioavailability and hinders its distribution throughout the body with the inherent difficulties to cross cellular membranes and attain the target sites^12^. The unmet need for new therapies targeting mitochondria stimulates the active search for new agents that can minimise mitochondria dysfunction. Within this framework, a number of mitochondria-targeted therapies have been developed, in particular, those using triphenylphosphonium (TPP) as carrier to deliver molecules to mitochondria^14–16^. Accordingly, it can be anticipated that the development of mitochondriotropic platforms for delivering dietary antioxidants is a rational strategy to prevent mitochondrial oxidative damage.

**Scheme 1.**
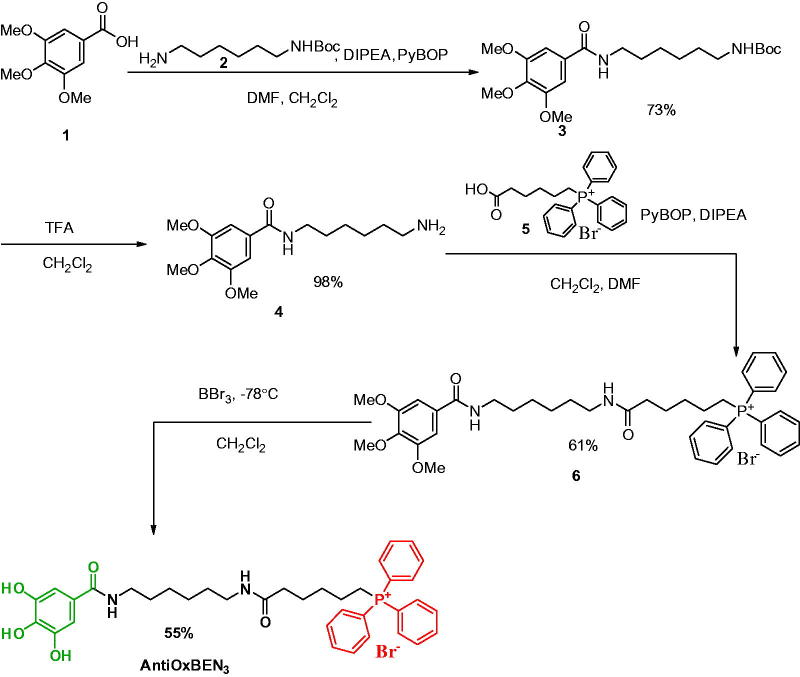
Synthetic strategy pursued for AntiOxBEN_3_ development.

As part of our long-term project related with the development of more effective antioxidants based on natural models, and guided by the data obtained so far^17^ ^,^ ^18^, we report here the development of a new mitochondriotropic antioxidant based on gallic acid, named as AntiOxBEN_3_ (Scheme 1), with the potential to inhibit the mitochondrial permeability transition.

## Materials and methods

*Reagents, general methods, and apparatus.* All reagents were purchased from Sigma-Aldrich (Barcelona, Spain) and used without additional purification. The solvents were pro-analysis grade and were acquired from Panreac (Lisbon, Portugal) and Sigma-Aldrich. Reaction progress was assessed by thin layer chromatography (TLC) analyses on aluminium silica gel 60 F254 plates (Merck, Darmstadt, Germany) in dichloromethane, ethyl acetate and dichloromethane/methanol, in several proportions. The spots were detected using UV detection (254 and 366 nm). Flash column chromatography was performed using silica gel 60 (0.040–0.063 mm) (Carlo Erba Reactifs – SDS, Val-de-Reuil, France). Following the workup, solvents were evaporated under reduced pressure in a Buchi Rotavapor (Buchi, New Castle, DE).

^1^H and ^13^C spectra NMR spectra were acquired at room temperature and recorded on a Bruker Avance III operating at 400 and 100 MHz, respectively. Chemical shifts are expressed in δ (ppm) values relative to tetramethylsilane (TMS) as internal reference and coupling constants (J) are given in Hz. Assignments were also made from DEPT (distortionless enhancement by polarisation transfer) (underlined values). Mass spectra (MS) were recorded on a Bruker Microtof (ESI) or Varian 320-MS (EI) apparatus and referred to as *m/z* (% relative) of important fragments.

The purity of the final products (>97% purity) was verified by high-performance liquid chromatography (HPLC) equipped with a UV detector.

### Chemistry

*Synthesis of tert-butyl (6–(3,4,5-trimethoxybenzamido)hexyl)carbamate (**3**)*. 3,4,5-trimethoxybenzoic acid (1, 500 mg, 2.3 mmol) was dissolved in DMF (3.9 ml) at 4 °C and then N,N-diethylpropan-2-amine (0.421 ml, 2.3 mmol) and PyBOP (1668 mg, 2.3 mmol) in CH_2_Cl_2_ (3.9 ml) were added. The mixture was kept in an ice bath and stirred for half an hour. After this period, tert-butyl (6-aminohexyl)carbamate (2, 0.529 ml, 2.3 mmol) was added and the mixture was allowed to warm up to room temperature. The reaction was kept with stirring for 18 h. The mixture was then diluted with dichloromethane (20 ml) and washed with saturated NaHCO_3_ solution (2 × 10 ml). The organic phase was dried over Na_2_SO_4_, filtered and concentrated under reduced pressure. The residue was purified by flash chromatography (50% AcOEt/petroleum ether), yield: 73%.

^1^H NMR (400 MHz, CDCl_3_): *δ* = 7.07 (2H, s, H5, H6), 6.55 (1H, s, H1′), 4.59 (1H, s, H8′), 3.91 (6H, s, 2XOCH_3_), 3.88 (3H, s, OCH_3_), 3.43 (2H, dd, *J* = 13.0, 6.9 Hz, H2′), 3.13 (1H, dd, *J* = 12.6, 6.2 Hz, H7′), 1.67–1.58 (2H, m, H3′), 1.53–1.32 (15H, m, H4′, H5′, H6′, NHCOOC(CH_3_)). ^13^C NMR (100 MHz, CDCl_3_): *δ* = 167.3 (CONH), 156.3 (NHCOOC(CH_3_)), 153.3 (C3, C5), 140.9 (C4), 130.4 (C1), 104.5 (C2, C6), 79.3 (NHCOOC(CH_3_)), 61.0 (OCH_3_), 56.4 (2XOCH_3_), 40.0 (C7′), 39.7 (C1′), 30.2 (C2′), 29.5 (C6′), 28.5 (NHCOOC(CH_3_)), 26.1 (C3′), 25.8 (C4′).

*Synthesis of N-(6-aminohexyl)-3,4,5-trimethoxybenzamide (**4**)*. The deprotection step was performed adding TFA (4 ml) to a solution of 3 (1 g, 2.4 mmol) in CH_2_Cl_2_ (8 ml). The reaction was stirred at room temperature for 1 h. After neutralisation with a saturated NaHCO_3_ solution, the organic phase was separated. The organic phase was dried over Na_2_SO_4_, filtered, and concentrated under reduced pressure. The residue was purified by flash chromatography (10% MeOH/CH_2_Cl_2_), yield: 98%.

^1^H NMR (400 MHz, MeOD): *δ* = 7.19 (2H, s, H2, H6), 3.89 (6H, s, 2XOCH_3_), 3.80 (3H, s, OCH_3_), 3.39 (1H, t, *J* = 7.1 Hz, H_2_), 2.99–2.90 (2H, m, H7), 1.77–1.55 (4H, m, H3, H6), 1.50–1.36 (4H, m, H4, H5). ^13^C NMR (100 MHz, MeOD): *δ* = 169.4 (CONH), 154.3 (C3, C5), 141.8 (C4′), 131.1 (C1), 105.9 (C2, C6), 61.2 (OCH_3_), 56.7 (2XOCH_3_), 40.8 (C7′), 40.6 (C1′), 30.2 (C6′, 28.4 (C3′), 27.4 (C4′), 27.0 (C5′).

*Synthesis of [5–(6-(3,4,5-trimethoxybenzamido)hexylamino)carbonylpentyl] triphenylphosphonium bromide (**6**)*. To a solution of compound **4** (689 mg, 2.2 mmol) in DMF (7.4 ml) at 4 °C N,N-diethylpropan-2-amine (0.476 ml, 2.7 mmol) and PyBOP (1572 mg, 2.7 mmol) in CH_2_Cl_2_ (7.4 ml) were added. The mixture was kept in an ice bath and stirred for half hour. After this period, compound **5** (1218, 2.7 mmol) was added and then the reaction was heated up to room temperature. The reaction was kept under stirring for 20 h. The mixture was then diluted with AcOEt (40 ml) and washed with saturated NaHCO_3_ solution (2 × 10 ml). The organic phase was dried over Na_2_SO_4_, filtered and concentrated under reduced pressure. The residue was purified by flash chromatography (10% MeOH/CH_2_Cl_2_), yield: 63%.

^1^H NMR (400 MHz, CDCl_3_): *δ* = 7.85–7.76 (3H, m, H4′′), 7.73–7.59 (12H, m, H2′′, H3′′, H5′′, H6′′), 7.12 (2H, s, H2, H6), 6.93 (1H, t, *J* = 5.7 Hz, H1′), 6.26 (1H, t, *J* = 5.7 Hz, H8′), 3.88 (6H, s, 2XOCH_3_), 3.85 (1H, s, OCH_3_), 3.39 (dd, *J* = 13.2, 6.7 Hz, 1H), 3.19–3.05 (4H, m, H7′, H14′), 2.14 (1H, t, *J* = 7.1 Hz, H10′), 1.69–1.26 (14H, m, H3′, H4′, H5′, H6′ H11′, H12′, H13′). ^13^C NMR (100 MHz, CDCl_3_): *δ* = 173.3 (C9′), 167.2 (PhCONH), 153.1 (C3, C5), 140.4 (C4), 135.4 (d, *J*_CP _= 2.9 Hz, C4′′), 133.4 (d, *J*_CP _= 9.9 Hz, C2′′,C6′′), 130.7 (d, J_CP _= 12.6 Hz, C3′′,C5′′), 130.4 (C1), 117.9 (d, *J*_CP _= 86.2 Hz, C1′′), 104.5 (C2, C6), 60.9 (OCH_3_), 56.4 (2XOCH_3_), 39.7 (C2′), 39.0 (C7′), 36.3 (C10′), 30.0 (C3′), 29.8 (C6′), 28.9 (d, *J* = 5.0 Hz, C12′), 26.6 (C4′), 25.9 (C5′), 24.9 (C11′), 22.3 (d, *J* = 43.5 Hz, C14′), 22.1 (d, *J* = 12.5 Hz, C13′) .

*Synthesis of [5–(6-(3,4,5-trihydroxybenzamido)hexylamino) carbonylpentyl]triphenylphosphonium bromide (AntiOxBEN_3_).* Compound **6** (1.0 g; 1.4 mmol) was dissolved in 7.6 ml of anhydrous dichloromethane. The reaction mixture was stirred under argon and cooled at a temperature below −75 °C. Boron tribromide (4.3 ml of 1 M solution in dichloromethane; 4.3 mmol) solution was added dropwise and the reaction was kept at −75 °C for 10 min. Once the addition was completed, the reaction was kept at −70 °C for 10 min and then allowed to warm to room temperature with continuous stirring for 12 h. Thereafter, the reaction was finished by slow addition of water (20 ml). After water removal, the resulting product was dissolved in methanol and dried over anhydrous Na_2_SO_4_, filtered, and the solvent evaporated. The residue was purified by flash chromatography (10% MeOH/CH_2_Cl_2_), yield: 55%.

^1^H NMR (400 MHz, MeOD): *δ* = 7.92–7.83 (3H, m, H4′′), 7.82–7.70 (12H, m, 12H, m, H2′′, H3′′, H5′′, H6′′), 6.83 (2H, s, H2, H6), 3.43–3.34 (2H, m, H14′), 3.33–3.25 (2H, m, H2′), 3.14 (1H, t, *J* = 6.9 Hz, H7′), 2.15 (1H, t, *J* = 7.0 Hz, H10′), 1.72–1.28 (14H, m, H3′, H4′, H5′, H6′ H11′, H12′, H13′). ^13^C NMR (100 MHz, MeOD): *δ* = 176.3 (C9), 170.5 (PhCOONH), 146.5 (C3, C5), 138.1(C4), 136.1 (d, *J* = 2.9 Hz, C4′′), 134.7 (d, *J*_CP _=10.0 Hz, C2′′, C6′′), 131.5 (d, *J*_CP _=12.6 Hz, C3′′, C4′′), 125.4 (C1), 119.7 (d, *J*_CP _= 86.3 Hz, C1′′), 107.8 (C2, C6), 40.8 (C2′), 40.5 (C7′), 36.2 (C10′), 30.9 (C3′), 30.8 (C6′), 30.2 (C4′), 29.9 (C5′), 27.4 (d, *J*_CP _= 2.5 Hz, C12′), 26.1 (C11′), 23.1 (d, *J*_CP _= 4.2 Hz, C13′), 22.6 (d, *J*_CP _= 51.3 Hz, C14′). ESI/MS *m/z* (%): 628 (M^+^+H-Br^−^, 38), 627 (M^+^−Br, 100), 556 (35), 547 (46). ESI/HRMS *m/z* calc. for C_37_H_44_N_2_O_5_P^+^ (M^+^−Br^−^): 627.2982; found 627.2970.

## Pharmacology

### Evaluation of AntiOxBEN3 functional mitochondrial toxicity profile

*Animals.* Male Wistar Han rats (10 weeks old) were housed in our accredited animal colony (Laboratory Research Center, Faculty of Medicine of University of Coimbra, Coimbra, Portugal). Animals were group-housed in type III-H cages (Tecniplast, Varese, Italy) and maintained in specific environmental requirements (22 °C, 45–65% humidity, 15–20 changes/hour ventilation, 12 h artificial light/dark cycle, noise level <55 dB) and with free access to standard rodent food (4RF21 GLP certificate, Mucedola, Settimo Milanese, Italy) and acidified water (at pH 2.6 with HCl to avoid bacterial contamination). The research procedure was carried out in accordance with European Requirements for Vertebrate Animal Research and approved by the animal welfare committee of the Center for Neuroscience and Cell Biology, University of Coimbra, Coimbra, Portugal. Further approval was obtained from the National Agency for Veterinary and Agriculture (DGAV), reference 0421/000/000/2016.

*Isolation of rat liver mitochondria.* Rat liver mitochondria (RLM) were prepared by tissue homogenisation followed by differential centrifugations in ice-cold buffer containing 250 mM sucrose, 10 mM HEPES (pH 7.4), 1 mM EGTA, and 0.1% fat-free bovine serum albumin^19^. After obtaining a crude mitochondrial preparation, pellets were washed twice and resuspended in washing buffer (250 mM sucrose and 10 mM HEPES, pH 7.4). Mitochondrial protein concentration was determined by the biuret assay using BSA (bovine serum albumin) as a standard^20^.

*Measurement of AntiOxBEN_3_ mitochondrial uptake.* The uptake of AntiOxBEN_3_ by energised RLM was evaluated by using an ion-selective electrode, according to previously established methods, which measures the distribution of tetraphenylphosphonium (TPP^+^). An Ag/AgCl_2_ electrode was used as reference. To measure AntiOxBEN_3_ uptake, RLM (0.5 mg protein/ml) were incubated with constant stirring, at 37 °C, in 1 ml of KCl medium (120 mM KCl, 10 mM HEPES, pH 7.2, and 1 mM EGTA). Five sequential 1 µM additions of AntiOxBEN_3_ were performed to calibrate the electrode response in the presence of rotenone (1.5 µM). Then succinate (10 mM) was added to generate ΔΨ and valinomycin (0.2 µg/ml) was added at the end of the experiment to dissipate ΔΨ. The mitochondrial accumulation ratio was calculated by the disappearance of AntiOxBEN_3_ from extra- to intramitochondrial medium assuming an intramitochondrial volume of 0.5 µ.l/mg protein and a binding correction expected for the mitochondrial uptake of TPP compounds^21^.

*Evaluation of AntiOxBEN_3_ effect on mitochondrial respiration.* Isolated RLM oxygen consumption was evaluated polarographically with a Clark-type oxygen electrode, connected to a suitable recorder in a 1 ml thermostated water-jacketed chamber with magnetic stirring, at 37 °C. The standard respiratory medium consisted of 130 mM sucrose, 50 mM KCl, 5 mM KH_2_PO_4_, 5 mM HEPES (pH 7.3), and 10 µM EGTA. Increasing concentrations of AntiOxBEN_3_ (2.5–10 µM) were added to the reaction medium containing respiratory substrates glutamate/malate (10 and 5 mM, respectively) or succinate (5 mM) and RLM (1 mg) and allowed to incubate for a 5 min period prior initiate the registration. State 2 was measured as the oxygen consumption measured during the 5 min incubation time with AntiOxBEN_3_. To induce state 3 respiration, 125 nmol ADP (when using glutamate/malate) or 75 nmol ADP (when using succinate) was added. State 4 was determined after cessation of ADP phosphorylation. Subsequent addition of oligomycin (2 µ.g/ml) inhibited ATP-synthase and resulted in oligomycin-resistant respiration. Finally, 1 µM FCCP was added to uncouple respiration. The presented results are means ± SEM of seven independent experiments.

*Evaluation of AntiOxBEN_3_ effect on mitochondrial transmembrane electric potential (ΔΨ).* Approximate values for mitochondrial transmembrane electric potential (ΔΨ) was estimated through the evaluation of fluorescence changes of safranine O (5 µM) and was recorded on a spectrofluorometer operating at excitation and emission wavelengths of 495 and 586 nm, with a slit width of 5 nm. Increasing concentrations of AntiOxBEN_3_ (2.5–10 µM) were added to the reaction medium (200 mM sucrose, 1 mM KH_2_PO_4_, 10 mM Tris (pH 7.4), and 10 µM EGTA) containing respiratory substrates glutamate/malate (5 and 2.5 mM, respectively) or succinate (5 mM) and RLM (0.5 mg in 2 ml final volume) and allowed to incubate for a 5 min period prior to recording, at 25 °C. In this assay, safranine (5 µM) and ADP (25 nmol) were used to initiate the assay and to induce depolarisation, respectively. Moreover, 1 µM FCCP was added at the end of all experiments to cause complete mitochondrial depolarisation. ΔΨ was calculated using a calibration curve obtained when RLM were incubated in a reaction medium mostly devoid of K^+^, containing 200 mM sucrose, 1 mM NaH_2_PO_4_, 10 mM Tris (pH 7.4), and 10 µM EGTA, supplemented with 0.4 µ.g valinomycin, as previous described^22^ ^,^ ^23^. The extension of fluorescence changes of safranine induced by ΔΨ was found to be similar in the standard and K^+^-free medium. “Repolarisation” corresponds to the recovery of apparent ΔΨ after the complete phosphorylation of ADP added. Lag phase reflects the time required to phosphorylate the added ADP. Values are means ± SEM of five independent experiments.

*Evaluation of AntiOxBEN_3_ iron chelating properties*. The assay was performed in ammonium acetate buffer (pH 6.7) using a solution of ammonium iron (II) sulphate in ammonium acetate as the source of ferrous ions. In each well, a solution of the test compound (100 µM) and ammonium iron (II) sulphate in ammonium acetate (20 µM) was added, incubated for 10 min and the absorbance read at 562 nm. An aqueous 5 mM solution of ferrozine was freshly prepared and then added to each well (96 µM final concentrations). After a new incubation at 37 °C during 10 min, the absorbance of [Fe(ferrozine)_3_]^2+^ complex was measured at 562 nm. EDTA was used as a reference. All compounds, including ferrozine, were tested at the final concentration of 100 µM. The absorbance of the first reading was subtracted from the final values to discard any absorbance due to the test compounds. Data are means ± SEM of three independent experiments and are expressed as Δabsorbance at 562 nm.

*Evaluation of AntiOxBEN_3_ effect on RLM lipid peroxidation*. The effect of AntiOxBEN_3_ on RLM lipid peroxidation was evaluated by measuring thiobarbituric acid reactive species (TBARS). RLM (2 mg protein/ml) were incubated in 0.8 ml medium containing 100 mM KCl, 10 mM Tris-HCl and pH 7.6, at 37 °C, supplemented with 5 mM glutamate/2.5 mM malate as substrates. RLM was incubated for a 5 min period with the different tested compounds (5 µM) after which mitochondria were exposed to oxidative stress condition by the addition of 100 µM FeSO_4_/500 µM H_2_O_2_/5 mM ascorbate for 15 min at 37 °C. After exposure to oxidative stress, 60 µl of 2% (v/v) butylated hydroxytoluene in DMSO was added, followed by 200 µl of 35% (v/v) perchloric acid and 200 µl of 1% (w/v) thiobarbituric acid. Samples were then incubated for 15 min at 100 °C, allowed to cool down and the supernatant transferred to a glass tube. After addition of 2 ml MiliQ water and 2 ml butan-1-ol, samples were vigorously vortexed for few seconds. The two phases were allowed to separate. The fluorescence of aliquots (250 µl) of the organic layer was analysed in a plate reader (λEx = 515 nm; λEm = 553 nm) for TBARS. Data are means ± SEM of three independent experiments and are expressed as % of control (control = 100%).

*Evaluation of AntiOxBEN_3_ effect on mitochondrial permeability transition pore opening.* Mitochondrial swelling was estimated by alterations of the light scattered from the mitochondrial suspension, Increasing concentrations of AntiOxBEN_3_ (2.5–10 µM) was added to the reaction medium (200 mM sucrose, 1 mM KH_2_PO_4_, 10 mM Tris (pH 7.4), 5 mM succinate and 10 µM EGTA supplemented with 1.5 µM rotenone) in the presence of RLM (1 mg) and allowed to incubate for a 5 min period prior to initiating the recording. The experiments were started by the addition of a suitable concentration of Ca^2+^ (15–50 µM), determined every day, and the absorbance at 540 nm monitored every minute for a 15 min period. Cyclosporin A (CsA) (1 µM), a mPTP de-sensitiser^24^, was added to confirm mPTP opening. The reaction was stirred continuously and the temperature maintained at 37 °C. Data are means ± SEM of three independent experiments and are expressed as Δabsorbance at 540 nm.

### Evaluation of AntiOxBEN_3_ cytotoxic and antioxidant cellular profile

*Cell culture conditions.* Hepatocellular carcinoma HepG2 cells (ECACC, Salisbury, UK) were cultured in high-glucose medium composed by Dulbecco′s modified Eagle′s medium (DMEM; D5648) supplemented with sodium pyruvate (0.11 g/l), sodium bicarbonate (1.8 g/l) and 10% foetal bovine serum (FBS) and 1% of antibiotic penicillin-streptomycin 100 × solution. Cells were maintained at 37 °C in a humidified incubator with 5% CO_2_.

*Cytotoxicity screening using sulforhodamine B assay.* After the treatment period, the sulforhodamine B (SRB) assay was used for cell mass determination, which is based on the measurement of cellular protein content. Briefly, after compound incubation, the medium was removed and wells rinsed with PBS (1X). Cells were fixed by adding 1% acetic acid in 100% methanol for at least 2 h at −20 °C. Then, this solution was discarded and the plates dried in an oven at 37 °C. Two hundred and fifty µl of 0.5% SRB in 1% acetic acid solution was added and incubated at 37 °C for 1 h. The wells were then washed to remove the excess of the dye with 1% acetic acid and dried. Then, 500 µ.l of Tris (pH 10) was added and the plates were stirred for 15 min. Finally, 200 µ.l of each supernatant was transferred in 96-well plates and optical density was measured at 540 nm.

*Antioxidant protective effect.* Cells were placed on 48-well plate (4 × 10^4^ cells/ml), cultured for 24 h before treatment and then were pre-incubated with AntiOxBEN_3_ (100 µM), a concentration in which cell mass was not affected, for 1 h. Cell were then exposed to oxidative stress by the addition of 250 µM FeSO_4_ or 250 µM H_2_O_2_ for 48 h. At the end of treatment time, the SRB assay was used for cell mass determination. Data are means ± SEM of six independent experiments and are expressed as percentage of control, which represents the cell mass without any treatment in the respective time point.

### Statistics

GraphPad Prism version 5.0 software (GraphPad Software, Inc., La Jolla, CA) was used for data analysis. All results were expressed as means ± SEM for the number of assays indicated in each experiment. Data were analysed by the student′s t-test for comparison of two means, and one-way ANOVA with Dunnet multiple comparison post-test to compare groups with one independent variable. Significance was accepted with *p < .05, **p < .01, ***p < .0005, ****p < .0001.

## Results and discussion

Research on mitochondriotropic antioxidants has been increasing over the last years. One viable and promising strategy involves the use of dietary polyphenolic antioxidants templates along with the chemical modulation of their properties, including mitochondrial targeting ability, efficacy, and toxicity^25^. In this context, some mitochondria-targeted polyphenolic-based molecules have been developed including MitoResveratrol, MitoCurcumin, and MitoQuercetin^26–28^. Despite the described antioxidant properties of the parent polyphenols, the new mitochondria-targeted derivatives have been shown to destabilise mitochondrial function exhibiting antiproliferative effects on different cell models, namely tumour cells^29–31^. Still, these works clearly show that mitochondrial targeting of polyphenols can be achieved, although in a disease context it is desirable that mitochondrial protection, and no toxicity, is attained.

In order to generate a mitochondrial-targeted gallic acid derivative with cytoprotective activity, AntiOxBEN_3_ was generated as a triparty entity having as a cap the gallic moiety, a peptide-like flexible spacer, and TPP as the ending group (Scheme 1). AntiOxBEN_3_ was synthesised following a four-step strategy in which trimethoxybenzoic acid 1 was linked to a monoprotected diamine 2 spacer to obtain the derivative 3. Compound **4** was obtained from compound **3** by a deprotection process in acid medium. Amine 4 was then coupled to the TPP cationic compound **5** by an amidation reaction in which the acylating agent was generated *in situ*. Then, compound **6** was demethylated using tribromide (BBr_3_) solution to obtain AntiOxBEN_3_. Globally, good to moderate yields have been obtained.

### AntiOxBEN_3_ mitochondrial uptake and functional mitochondrial toxicity profile

**Figure 1.**
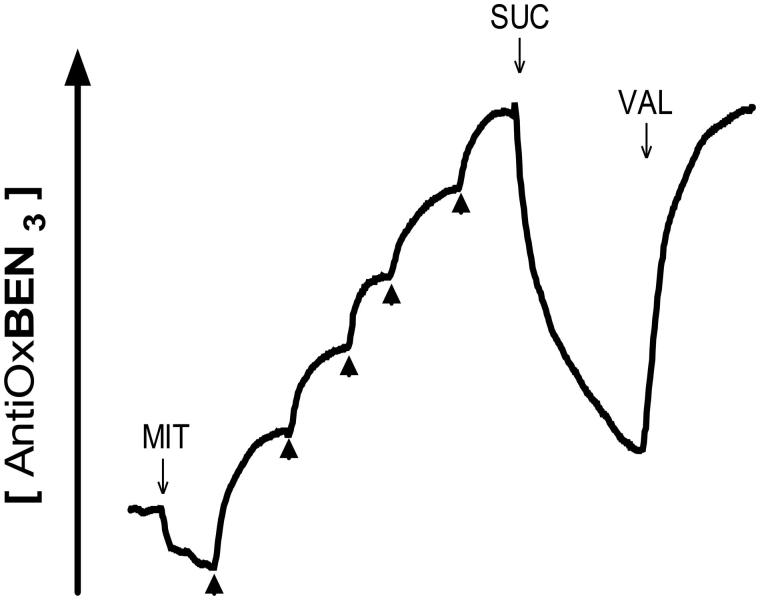
AntiOxBEN_3_ uptake by energised rat liver mitochondria measured using a TPP-selective electrode. MIT, mitochondria; SUC, succinate; VAL, valinomycin.

The next step was to evaluate AntiOxBEN_3_ mitochondrial uptake by measuring its accumulation in isolated RLM in response to the membrane electric potential (ΔΨ)^32^. In the presence of rotenone, ΔΨ was generated by the addition of Complex II substrate succinate (10 mM), leading to a decrease in the extramitochondrial compound concentration. The accumulated AntiOxBEN_3_ was extruded from mitochondria once ΔΨ was abolished by the K^+^-ionophore valinomycin (VAL) (Figure 1). The ΔΨ generated by RLM resulted into accumulation of approximately 5000–fold within the mitochondrial matrix. Similarly to other lipophilic antioxidants containing a TPP cation^17^ ^,^ ^18^ ^,^ ^21^, AntiOxBEN_3_ was able to penetrate in mitochondria driven by the ΔΨ, accumulating up to several hundred-fold, increasing significantly the concentration and potency of the targeted compound.

Isolated rat hepatic mitochondrial fractions were also used to detect direct toxic effects of AntiOxBEN_3_ on the bioenergetics apparatus. Similarly to previous studies^17^ ^,^ ^18^, the effect of AntiOxBEN_3_ on mitochondrial bioenergetic apparatus was evaluated at three different concentrations (2.5, 5, and 10 µM) by measuring O_2_ consumption and approximate ΔΨ. In addition, mitochondrial functionality parameters (RCR, respiratory control ratio and ADP/O ratio, which measure the ADP phosphorylation efficiency) were evaluated.

Hepatic mitochondrial fractions were energised with Complex I or Complex II substrates, developing an apparent ΔΨ ≈ 230 mV or ≈ 186 mV (negative inside), during glutamate/malate- and succinate-energisation, respectively (Table 1). Despite AntiOxBEN_3_ mitochondriotropic mechanism, it is important to note that the alterations caused by the compound on the ΔΨ values measured were not statistically significant.

**Table 1.** Effect of AntiOxBEN_3_ on mitochondrial bioenergetics: mitochondrial respiratory control ratio (RCR); ADP phosphorylation efficiency (ADP/O); and approximate transmembrane electric potential (ΔΨ).

			AntiOxBEN_3_		
	Mitochondrial Bioenergetics	Control	2.5 µM	5 µM	10 µM
Glut/Mal	Maximum potential (app. ΔΨ in − mV)	229.8 ± 17.4	221.1 ± 20.2	221.4 ± 22.6	227.5 ± 26.3
	RCR	7.3 ± 0.6	3.9 ± 0.5**	3.9 ± 0.6**	3.07 ± 0.6^****^
	ADP/O	2.6 ± 0.1	2.3 ± 0.2	2.3 ± 0.1	2.0 ± 0.2*
Succinate	Maximum potential (app. ΔΨ in − mV)	186.1 ± 6.6	203.6 ± 16.6	205.3 ± 19.4	207.9 ± 19.3
	RCR	4.1 ± 0.3	4.1 ± 0.5	4.3 ± 0.7	3.9 ± 0.4
	ADP/O	1.5 ± 0.1	1.6 ± 0.1	1.6 ± 0.1	1.7 ± 0.1

Effect of AntiOxBEN_3_ on approximate ΔΨ, RCR and ADP/O of energised RLM (5 mM glutamate/2.5 malate or 5 mM succinate). Values are means ± SEM of five independent experiments. Statistically significant compared with control using Student’s two tailed t-test. Significance was accepted with *p < .05, **p < .01, ****p < .0001.

**Figure 2.**
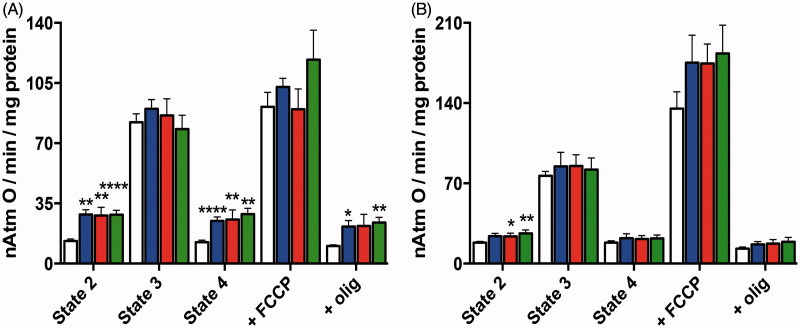
Effect of AntiOxBEN_3_ on RLM respiration supported by (A) 10 mM glutamate +5 mM malate or (B) 5 mM succinate. The white bars refer to control, while blue, red and green bars refer to experiments where RLM were pre-incubated with AntiOxBEN_3_ (2.5, 5, and 10 μM, respectively). Olig, oligomycin. The presented results are means ± SEM of seven independent experiments. *p < .05, **p < .01, ****p < .0001 vs. control.

Hepatic mitochondrial fractions were similarly energised in order to detect direct effects of AntiOxBEN_3_ on mitochondrial O_2_ consumption. Respiratory rates characteristic of state 2, state 3, state 4, oligomycin-resistant respiration and FCCP-stimulated respiration are shown in Figure 2. The respiratory control ratio (RCR, state 3/state 4 respiration), which gives insight on oxidative phosphorylation coupling, was of 7.3 ± 0.6 and 4.1 ± 0.3 for the control experiments when glutamate-malate and succinate were used as respiratory substrates, respectively. ADP/O was 2.6 ± 0.1 and 1.5 ± 0.1 when glutamate-malate and succinate, respectively, were used as respiratory substrates (Table 1). Although AntiOxBEN_3_-induced alterations on mitochondrial respiration supported by the two substrates followed the same general tendency, these changes were more pronounced when Complex I substrates were used. AntiOxBEN_3_ caused a concentration-dependent marked increase in state 2, state 4 and oligomycin-resistant respiration (Figure 2). The overall consequence was a significant decrease in the RCR. The ADP/O was also significantly affected with 10 µM AntiOxBEN_3_ (Table 1). This new mitochondriotropic agent appeared to increase the mitochondrial inner membrane permeability to protons, or to other cations, a process that may occur through the induction of a membrane disturbance. Although several mitochondria-targeted rhodamine cationic derivatives presented uncoupling effect^33^, our data argue against a protonophoretic activity exerted by AntiOxBEN_3_, since this mitochondria-targeted antioxidant had a negligible effect on the apparent ΔΨ measured (Table 1). These types of compounds have a strong tendency to adsorb as a monolayer onto the surface of phospholipid bilayers and the TPP component is always found at the same position in the potential energy well on the membrane surface, whereas the hydrophobic alkyl chain is usually inserted into the hydrophobic core of the membrane. Moreover, the high volume of matrix-facing mitochondrial membrane relative to that of the matrix means that a very large proportion of the TPP cation within mitochondria is membrane-bound^7^, which may explain the increase of mitochondrial inner membrane permeability to protons.

Mitochondria-targeted antioxidants containing the TPP^+^ moiety can freely pass through cellular phospholipid bilayers, with the extent of anchoring being mainly dependent upon their hydrophobicity. Consequently, it is somehow expected that AntiOxBEN_3_ would exhibit similar cytotoxicity towards hepatocarcinoma cells as another type of mitochondriotropic hydroxybenzoic acid derivatives^34^ ^,^ ^35^. Surprisingly, AntiOxBEN_3_ was less toxic regarding mitochondrial functional end-points in isolated liver fractions in the same range of concentrations tested, meaning that the type (ester vs. amide) and length of the linker may also play a role on hydroxybenzoic acid derivatives induced-toxicity. Recent works showing targeting of different polyphenols to mitochondria reported that their mechanism of action involves the destabilisation of ΔΨ and consequent induction of mPTP opening^34^ ^,^ ^36^. Although AntiOxBEN_3_ also addressed gallic acid to mitochondria, antioxidant properties of the precursor were maintained and mitochondrial function was not visibly affected, which is clearly an advantage over these recent works.

### AntiOxBEN_3_ iron chelation properties

**Figure 3.**
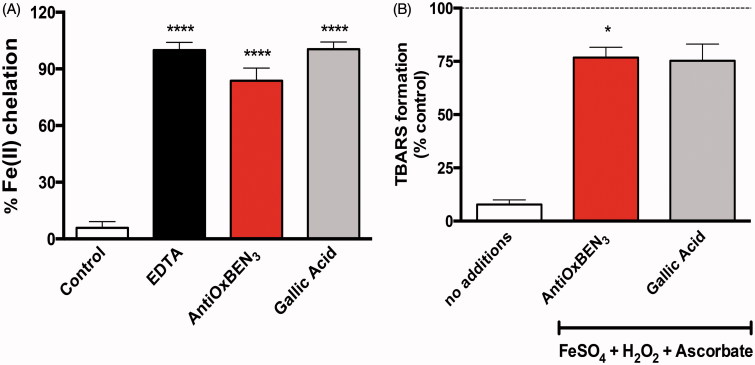
AntiOxBEN_3_ antioxidant properties. (A) AntiOxBEN_3_ iron chelation properties, EDTA (chelating agent) was used as reference. Data are means ± SEM from three independent experiments and are expressed as % of Fe(II) chelation. (B) AntiOxBEN_3_ effect on mitochondrial lipid peroxidation. Data are means ± SEM from three independent experiments and are expressed as % of control. ****p < .0001 vs. control (A), *p < .05 vs. no additions (B).

As iron overload and loss of iron homeostasis are associated with oxidative stress^37^, and ultimately to mitochondrial dysfunction, the AntiOxBEN_3_ iron chelating properties were evaluated. Data show that AntiOxBEN_3_ can chelate ferrous iron, as observed for the significant decrease in [Fe(ferrozine)_3_]^2+^ complex formation. Still, EDTA, a well-known metal chelator, was the best chelating agent tested (Figure 3(A)), as the binding constant of EDTA for its complex with iron is higher than that of phenolic acids^38^. Most important, the TPP cation and the alkyl spacer did not have a relevant effect on AntiOxBEN_3_ chelation properties, when compared to gallic acid alone. AntiOxBEN_3_ metal chelation properties, which are similar to that presented by gallic acid, can be ascribed to the presence of the pyrogallol system and are likely involved in their antioxidant mechanism. As, gallic type systems have intrinsic metal chelating properties^39^, and this motif was not altered in AntiOxBEN_3_, one can consider that it is the moiety responsible for the observed iron chelation and antioxidant activities.

### AntiOxBEN3 effects on lipid peroxidation

The enrichment of mitochondrial membranes in polyunsaturated fatty acids, and their proximity to ROS production sites, makes mitochondria particularly vulnerable to lipid peroxidation. Therefore, the antioxidant action of AntiOxBEN_3_ antagonising lipid peroxidation was evaluated. AntiOxBEN_3_ prevented lipid peroxidation stimulated by H_2_O_2_/FeSO_4_/ascorbate system, assessed as TBARS production in RLM (Figure 3(B)). Similarly to gallic acid, that prevents lipid peroxidation due to its antioxidant and anti-lipoperoxidative properties^40^ ^,^ ^41^, AntiOxBEN_3_ prevented lipid peroxidation likely through its direct radical scavenging activity, although its direct iron-chelation properties cannot be discarded. In fact, AntiOxBEN_3_ can chelate the ferrous iron present in solution, which is maintained in this form by the presence of ascorbate in the oxidative system.

### AntiOxBEN3 effects on mitochondrial permeability transition pore

Mitochondrial permeability transition pore (mPTP) opening is usually linked to mitochondrial dysfunction, as it results in a solute exchange between mitochondrial matrix contents and the surrounding cytoplasm, and is connected to mitochondrial depolarisation, cessation of ATP synthesis, Ca^2+^ release, pyridine nucleotide depletion, inhibition of respiration and ultimately to organelle swelling and membrane rupture^42^. mPTP opening is involved in the toxicity process of different xenobiotics^43^ ^,^ ^44^ and in different pathologies, which ultimately result in cell damage and death^45^ ^,^ ^46^.

**Figure 4.**
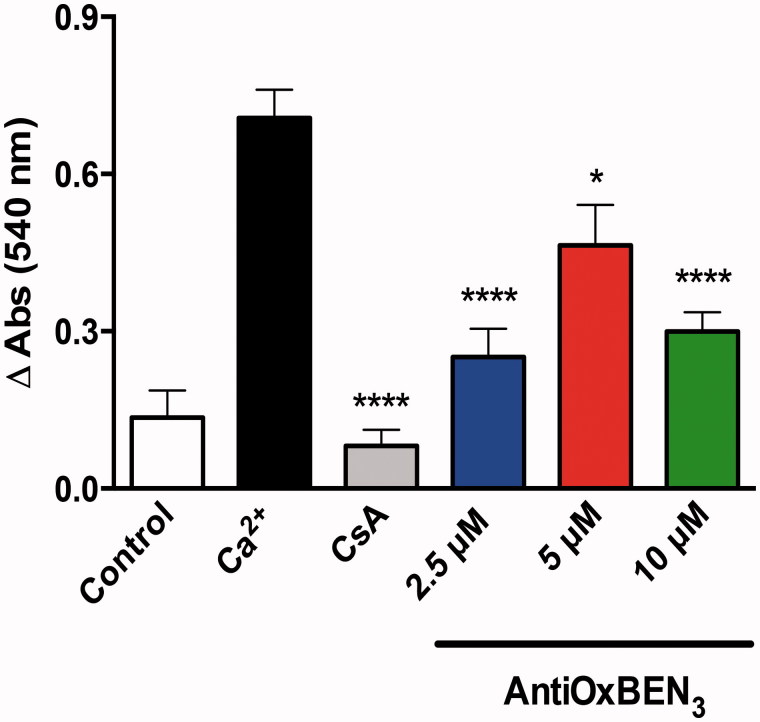
AntiOxBEN_3_ effects on mitochondrial swelling resulting from induction of the mitochondrial permeability transition pore (mPTP) opening. Data are means ± SEM from three independent experiments and are expressed as Δabsorbance at 540 nm. *p < .05, ****p < .0001 vs. Ca^2+^.

As mPTP opening can be induced, among other factors, by calcium (Ca^2+^) overload and excessive ROS production, we evaluated AntiOxBEN_3_ effects on calcium-induced mPTP opening. CsA, a mPTP de-sensitiser^24^, was added to confirm that the mitochondrial swelling observed resulted from mPTP induction. Remarkably, for all AntiOxBEN_3_ tested concentrations, no mPTP inducing effect was observed. In opposition, AntiOxBEN_3_ showed concentration-dependent inhibitory effects (Figure 4) similarly to CsA. Increased mitochondrial membrane permeability due to opening of the mPTP may be greatly enhanced by adenine nucleotide depletion, calcium influx, elevated phosphate, and oxidative stress^47^. AntiOxBEN_3_ protective effects may be related with its antioxidant activity, interference with one of the mPTP components or through the chelation of calcium ions. The capability of gallic acid chelate zinc, calcium, and magnesium metals and the stability of such complexes confirmed the evidence that phenolic chelators possess chelating power either for mono and divalent metals^48^, although it is possible that in our present case, and based on the stoichiometry of the complexation reactions, some free calcium may still be available. Recently, it was demonstrated that gallic acid prevented mitochondrial swelling induced by different stimuli independent of calcium overload, suggesting that it act as a genuine inhibitor of mPTP and not by affecting mitochondrial calcium loading^49^. The ATP synthase has recently been proposed as the molecular component of the mPTP^42^. Inhibitory effects of some polyphenols on the ATP synthetase/ATPase activities were extensively reviewed^50^ ^,^ ^51^. Although catechin gallates (flavonoid esters of gallic acid) were mentioned, no references to gallic acid is mentioned. In a far-stretch assumption, Nanjundaiah et al. reported the gastroprotective effect of ginger rhizome, mainly due to the role of gallic and cinnamic acid anti-oxidative mechanism and inhibition of H^+^,K^+^-ATPase of H. pylori^52^. Yet, future mechanistic studies must be performed to understand how AntiOxBEN_3_ desensitises mPTP, including possible effects on the ATP synthase.

### Cytotoxicity of AntiOxBEN3 on HepG2 cells

**Figure 5.**
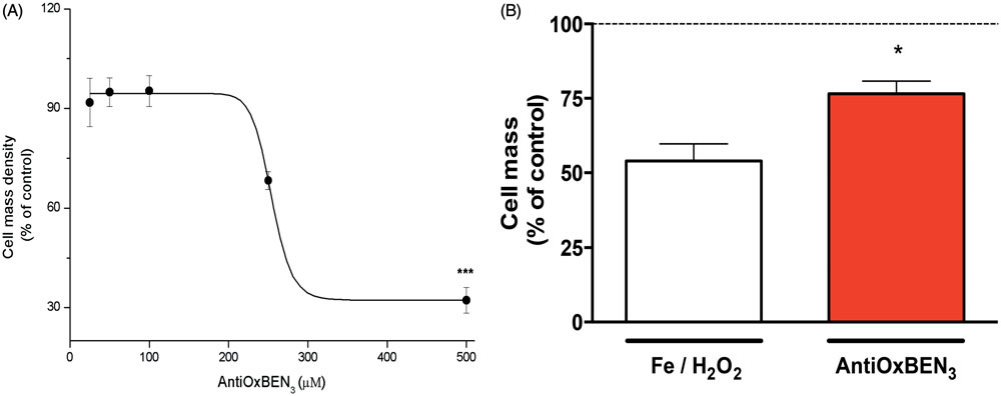
AntiOxBEN_3_ cytotoxicity and antioxidant outline on human hepatocellular carcinoma cells (HepG2). (A) Cytotoxicity profile on HepG2. (B) Antioxidant profile on HepG2 using iron and hydrogen peroxide as oxidant stressors. Data are means ± SEM from five independent experiments and are expressed as % of control. *p < .05 vs. Fe/H_2_O_2_.

AntiOxBEN_3_ cytotoxic profile was assessed^19^ on a human hepatocellular carcinoma cell line (HepG2), an *in vitro* system often used in toxicological studies. From the cytotoxicity data (IC_50 _=254 ± 32 µM), it can be concluded that AntiOxBEN_3_ presented low cellular toxicity (Figure 5(A)) having a promising safety margin for clinical use. Concurrently, a new mitochondriotropic compound based on gallic acid was developed by Jara et al.^34^ aiming to disrupt mitochondrial functioning in tumour cells by a mechanism similar to the one proposed for gallic acid ester derivatives. Antioxidants can be seen as a double-edged sword as they can also act as pro-oxidants in a diversity of systems based on its structure, concentration, and cellular redox context^53^. The TPP-gallic acid ester derivative (TPP + C12) is very toxic for different tumour cell lines (IC_50 _= 1 µM)^34^, while AntiOxBEN_3_ (having 12 carbons and two peptide bonds) showed lower toxicity towards HepG2 cells (IC_50 _= 250 µM). The type of spacer linking gallic acid and the TPP moiety differs between these two mitochondria-targeted molecules, suggesting that presence of an ester bond potentiate cytotoxicity effects. Moreover, the previously described mitochondriotropic agents based on gallic acid are toxic and can be easily hydrolysed by esterases limiting the administration route and biological usefulness.

### AntiOxBEN3 antioxidant effects on HepG2 cells

HepG2 cells were then incubated with different inducers of oxidative stress (250 µM FeSO_4_ and 250 µM H_2_O_2_). The oxidant stressor resulted into a significant inhibition of cell proliferation when compared with control. Yet, pre-treating cells with AntiOxBEN_3_ significantly prevented iron- and hydrogen peroxide-induced HepG2 cytotoxicity (Figure 5(B)). From this and previous data, esterification of carboxylic group and length of the linker seems to potentiate hydroxybenzoic acids cytotoxicity^34^ ^,^ ^35^ while peptide-like bond, present in AntiOxBENs, potentiate antioxidant activity^17^. Jara et al. reported that TPP-gallic acid ester derivatives (TPP + C8–12) presented cytotoxic effects on different tumour cell lines at the low micromolar range (1–10 µM)^34^, although AntiOxBEN_3_ (100 µM) presented effective antioxidant activity towards Fe/H_2_O_2_ (250 µM/250 µM) in a hepatocarcinoma cell line. Furthermore, Jara et al. reported that TPP + C8–12 induced mPTP opening in cells^34^, while AntiOxBEN_3_ prevented the Ca^2+^-induced mPTP opening in isolated mitochondrial fractions. Once again, only the type of spacer linking gallic acid and the TPP moiety differs between these two mitochondria-targeted molecules. Herein, we pointed out that the driving force on cytotoxic effects of mitochondria-targeted gallic acid derivatives may not be the aromatic ring pattern substitution. Actually, in the AntiOxBEN_3_ molecule, the spacer is linked to gallic acid and TPP moiety by two peptide-like bonds making this mitochondriotropic antioxidant less toxic and more stable on biological systems.

## Conclusion

This work highlights the successful development of a new mitochondriotropic antioxidant based on gallic acid that efficiently transports gallic acid to mitochondria without disturbing mitochondrial function and with distinct iron-chelation and antioxidant properties overcoming gallic acid bioavailability drawbacks. AntiOxBEN_3_ low cytotoxicity profile allows its use in the prevention of mitochondrial oxidative damage and in the regulation of oxidative stress pathways. Additionally, it was shown that this type of mitochondriotropic antioxidants can prevent calcium-dependent mPTP opening. So, AntiOxBEN_3_ can be considered a promising lead compound for the development of a new class of mPTP inhibitors to be used as mPTP de-sensitiser for basic research or clinical applications or undergo an optimisation programme from which a new drug based on gallic acid can emerge for therapeutic application in mitochondria dysfunction-related disorders.
